# jCompoundMapper: An open source Java library and command-line tool for chemical fingerprints

**DOI:** 10.1186/1758-2946-3-3

**Published:** 2011-01-10

**Authors:** Georg Hinselmann, Lars Rosenbaum, Andreas Jahn, Nikolas Fechner, Andreas Zell

**Affiliations:** 1 University of Tübingen, Center for Bioinformatics Tübingen (ZBIT), Sand 1, 72076 Tübingen, Germany

## Abstract

**Background:**

The decomposition of a chemical graph is a convenient approach to encode information of the corresponding organic compound. While several commercial toolkits exist to encode molecules as so-called fingerprints, only a few open source implementations are available. The aim of this work is to introduce a library for exactly defined molecular decompositions, with a strong focus on the application of these features in machine learning and data mining. It provides several options such as search depth, distance cut-offs, atom- and pharmacophore typing. Furthermore, it provides the functionality to combine, to compare, or to export the fingerprints into several formats.

**Results:**

We provide a Java 1.6 library for the decomposition of chemical graphs based on the open source Chemistry Development Kit toolkit. We reimplemented popular fingerprinting algorithms such as depth-first search fingerprints, extended connectivity fingerprints, autocorrelation fingerprints (e.g. CATS2D), radial fingerprints (e.g. Molprint2D), geometrical Molprint, atom pairs, and pharmacophore fingerprints. We also implemented custom fingerprints such as the all-shortest path fingerprint that only includes the subset of shortest paths from the full set of paths of the depth-first search fingerprint. As an application of jCompoundMapper, we provide a command-line executable binary. We measured the conversion speed and number of features for each encoding and described the composition of the features in detail. The quality of the encodings was tested using the default parametrizations in combination with a support vector machine on the Sutherland QSAR data sets. Additionally, we benchmarked the fingerprint encodings on the large-scale Ames toxicity benchmark using a large-scale linear support vector machine. The results were promising and could often compete with literature results. On the large Ames benchmark, for example, we obtained an AUC ROC performance of 0.87 with a reimplementation of the extended connectivity fingerprint. This result is comparable to the performance achieved by a non-linear support vector machine using state-of-the-art descriptors. On the Sutherland QSAR data set, the best fingerprint encodings showed a comparable or better performance on 5 of the 8 benchmarks when compared against the results of the best descriptors published in the paper of Sutherland et al.

**Conclusions:**

jCompoundMapper is a library for chemical graph fingerprints with several tweaking possibilities and exporting options for open source data mining toolkits. The quality of the data mining results, the conversion speed, the LPGL software license, the command-line interface, and the exporters should be useful for many applications in cheminformatics like benchmarks against literature methods, comparison of data mining algorithms, similarity searching, and similarity-based data mining.

## Background

The decomposition of a chemical graph into a list of features is a convenient way to assess the similarity between chemical compounds by comparing the resulting lists of features. Such representations are also called chemical fingerprints [1]. These encodings are important for data mining applications like similarity-based machine learning approaches or similarity searches [2].

The goal of this work is to introduce an open source molecular fingerprinting library for data mining purposes which provides exact definitions of its fingerprinting algorithms. The algorithms can be parametrized with various options to adapt the encodings, for example, by applying a custom labeling function or by altering the search depth parameter. Additionally, the library can be used as a basis for new implementations. It is based on the Chemistry Development Kit [3], which also provides several fingerprints in its API. However, there are several differences. The first aim of jCompoundMapper is to focus on the exact definition of its encodings, which is crucial to describe the features in data mining experiments. The second aim is to provide the functionality to export the fingerprints or pairwise similarity matrices to formats of popular machine learning toolboxes. A label or property of an input compound to be trained by a machine learning algorithm can be included.

Most fingerprint algorithms rely on either the geometrical or the topological distance between the atoms of a structure. The topological information is stored in the all-shortest path matrix, which encodes the minimum topological distance between two atoms (vertices) by the shortest path using the bonds (edges). Organic compounds are usually weakly connected because the number of covalent bonds (vertex degree) of an organic molecule is limited. In contrast, the geometry of a structure can be interpreted as a fully connected graph. The complexity of both approaches can reduced by limiting the search depth for topological fingerprints or by introducing a distance cut-off for geometrical fingerprints.

jCompoundMapper offers a variety of topological (e.g. radial atom environments [4], extended connectivity fingerprints [5], depth-first search fingerprints [6], or auto-correlation vectors [7]) and geometrical (e.g. two-point and three-point encodings [8,9] or geometrical atom environments [10]) fingerprints. If applicable, it allows for a parameterization of an encoding, such as the search depth, the distance cut-off, the geometrical scaling factor, the atom typing scheme, or the hash space.

After the feature generation step, the list of features can be mapped to a vectorial format. One possibility is to encode a set of features as a hashed fingerprint. Here, a unique identifier of a feature is used to initialize a pseudo random number generator which produces numbers in $[0,h]\in {\mathbb{N}}^{+}$, where *h *is the maximum size of the hash space. Thus, the dimensionality of the original feature space can be considerably reduced. For example, the Fingal fingerprint [11], uses the cyclic redundancy check algorithm to generate seeds for the hashing of chemical graph patterns. For an introduction into hashed fingerprints, please refer to the review by Brown [1]. Another strategy reserves fixed bit positions in a vector for specific feature types, like patterns obtained at a certain parameter (such as depth or distance) with a limited number of possible combinations. The definition of the CATS2D [7] vector is an example for this approach.

jCompoundMapper supports native formats of common open source machine learning libraries. The exporters can be used to write feature maps to comma-separated format, LIBSVM [12] format (sparse and matrix), and WEKA ARFF [13]. Therefore, various data mining libraries can be directly applied on the output files. Furthermore, the library provides efficient data structures to compare sets of features in the case that the computation of a similarity matrix is required.

The quality of the encodings was compared on QSAR and toxicity benchmark problems in the results section. First, we conducted experiments using the support vector regression of LIBSVM on the well-known Sutherland QSAR benchmark set [14]. Second, we used a large Ames toxicity classification benchmark [15] and LIBLINEAR [16] to evaluate the performance using binary hashed sparse fingerprints. On the Sutherland data sets, the averaged squared correlation of the all-shortest-path and the atom triplet fingerprint was at least 5% better on ACHE than the best encoding given by Sutherland et al. On BZR and DHFR, the all-shortest path fingerprint achieved a squared correlation of 0.57 and 0.76 respectively. The performance was comparable on two data sets. On the remaining three data sets, the best encoding was more than 5% worse than the results of the best encoding published by Sutherland et al. On the Ames toxicity data set, the implementation of the extended connectivity fingerprint achieved an AUC ROC performance of 0.87, which is comparable to the performance by a non-linear support vector machine trained on state-of-the-art descriptors. Nevertheless, the goal was not an exhaustive comparison but to show that the implementations are able to obtain similar results when compared against literature results. jCompoundMapper features a command-line interface but can also be used as a Java API. It depends solely on open source libraries and is licensed under the LPGL. The source code and an executable is available at Sourceforge.

The library originated from various implementations of literature fingerprints and descriptors used in comparison studies. The encodings were employed either as part of a new approach or as a reference method [17-21].

To sum up, jCompoundMapper is an open source library for the encoding of chemical graphs as fingerprints. It can be used from the command-line interface or as a Java API. Hence, a further use in applications, like in KNIME http://www.knime.org nodes, is possible. The overall performance of the fingerprints in machine learning experiments indicates that structured-based models of reasonable quality can be obtained.

## Methods

### Prerequisites

#### Notation

The binned geometrical distance matrix *G_ij _*encodes the spatial distances between two heavy atoms. The topological distance matrix *T_ij _*encodes the shortest topological distance between atoms *i *and *j*. The labeling function $l(a_{i})\to {\hat{a}}_{i}$ types an atom according to a specific labeling scheme. The maximum distance allowed between two atoms (geometrical or topological) *d *defines a distance cut-off for features, all features with *g_ij _*>*d *or *t_ij _*> d are omitted. A labeled path *p *is a sequence of atoms connected by bonds $p=({\hat{a}}_{0},\;b_{0},{\hat{a}}_{1},\;b_{1},\;\ldots ,\;b_{d-1},{\hat{a}}_{d})$, where bond *b_i _*connects ${\hat{a}}_{i}$ with ${\hat{a}}_{i+1}$. *p*_*ij *_denotes a path connecting the *i*th atom with the *j*th atom. The depth *d *for topological patterns is the maximum number of bonds allowed for connecting the first atom with the last atom. Analogously to the definition of topological paths, a geometrical pattern must consist of different atoms, i.e. for two atoms *a*_*i*_, *a*_*j *_it holds that *i *≠ *j*. Finally, *a *⊕ *b *is defined as the concatenation of alphanumerical string symbols separated by an unique delimiter. In the following, we assume a hydrogen-depleted molecular graph *C *with *n *atoms.

An encoding algorithm *F *has the form

(1) $$F(C)\to X=\left\{f_{1},\;f_{2},\;\ldots ,\;f_{m}\right\}$$

where an encoding algorithm *F *maps some compound *C *to a set of features *X*. *m *depends, with the exception of fixed-vector fingerprints, on *C*. A feature *f *has an unique id∈ℕ and a string representation *f.nom. f.id *does not necessarily depend on *f.nom*. However, in most cases it is convenient to use a hash code of the string representation of a feature.

#### Fundamental Matrices

The geometrical distance matrix *G_ij _*is computed as the matrix of binned Euclidean distances in Ångstrom between the three-dimensional coordinates of all atom coordinates *a_i_*.*c *∈ ℝ^3^, multiplied by a scaling factor *s *∈ ℝ^+^. The scaling factor *s *influences the resolution of the geometry and should be chosen according to the size of the compounds. The entry *i*, *j *(*g_ij_*) in the matrix is calculated as follows

(2) gij=|(ai.c−aj.c) · s|.

The computation time for the geometrical distance matrix is quadratic. The binning of the real-valued geometrical distance is important to produce discrete features *f_x_*, *f_y_*, which can be compared by the Dirac function

(3) $$\delta (f_{x},\;f_{y})\text{ :}=\{\begin{array}{cc} 1, & \text{if}\;f_{x}=f_{y}\\ 0, & \text{else} \end{array}.$$

The topological distance matrix is defined as *T_ij_*. The element *i, j *contains the shortest path between the *i*th and the *j*th atom (*t_ij_*). *t_ij _*is computed by the Floyd-Warshall algorithm. Therefore, the computation time for the matrix is *O*(*n*^3^).

#### Feature Extraction

The molecular similarity is based on the numerical identifiers *f_x_.id *of a feature *x*. Two features are regarded as equal if *f_x_.id *= *f_y_.id*. In all implementations the features of a compound *C *are distinguishable by recurrence, which means that we include a feature if the id of a feature is different from the previously extracted features. If a feature with the same id is generated again, the count for the feature is incremented. All atom pair encodings are extracted by regarding the upper half of the distance matrix only. For each atom pair, the string representation is generated in both reading directions. Only the version with the greater hash code is included in the final set of descriptors.

The modified depth-first search applied in this library generates all possible paths originating from a root atom. Therefore, the feature space can be approximated by an *m*-ary tree and is therefore *O*(*nm^d^*), where *n *is the number of heavy atoms and *m *the number of children in an *m*-ary tree, *d *is the depth of the tree. In organic compounds, every atom has at most 4 neighbors (*m *= 4 - 1 because one of the neighboring bonds has already been visited). Thus, the hypothetical worst case has a complexity of *O*(*n*3*^d^*) at a search depth of *d*. If we assume an average branching factor *α*, which is slightly above 1 for organic compounds [6], the depth-first search has a complexity of *O*(*nα^d^*). The average branching factor depends on the average degree of a vertex, which is about 2 in organic molecules. We define *DFS*(*a_i_*, *d*) as the set of all possible paths originating from a root atom *a_i _*with a depth up to *d*.

For some of the definitions, we defined a *can *function that maps a set of features to a single canonical pattern. In an implementation this function can be realized by first sorting the patterns, which is possible if a natural order can be defined on the features. Then, the list of sorted patterns can be merged to a single canonical representation.

#### Atom Types and Pharmacophore Types

jCompoundMapper applies the standard atom types and ring detection algorithms implemented in the CDK. There are various typing schemes for small drug-like compounds described in the literature. In the current version, jCompoundMapper features the following typing schemes as labeling function $l(a_{i})\to {\hat{a}}_{i}$:

1. Element symbol (e.g. C, O, N, ...)

2. CDK atom types (e.g. C.sp2, O.minus, N.amine, ...)

3. Element plus the number of neighboring heavy atoms (e.g. C.2, O.1, N.2, ...)

4. Element plus ring type plus the number of neighboring heavy atoms (e.g. C.r.2, C.a.2, O.1, N.2, ...) where *r *is an arbitrary ring, and *a *is an aromatic system. If *a_i _*is not contained in a ring, no ring type is set. The precedence is *a *>*r*.

5. Daylight-Invariants (plus optional ring flag) have the following properties, separated by a dot: Atomic number, number of heavy atom neighbors, valency minus the number of connected hydrogens, atomic mass, atomic charge, number of connected hydrogens, and a flag if the atom is member of at least one ring. (e.g. 6.2.3.12.0.1.1 for a carbon in a benzole ring)

The following listing of potential pharmacophore points (PPPs) was published by Renner et al. [7] for the CATS autocorrelation descriptors. If PPP atom types are needed, this list is parsed and matched with the structure using the CDK SMARTS matcher or specially implemented graph searches.

1. Hydrogen-bond donor (D): [#6H] oxygen atom of an OH-group; [#7 H,#7H2] nitrogen atom of an NH or NH_2 _group

2. Hydrogen-bond acceptor (A): oxygen atom [#6]; [#7H0] nitrogen atom not adjacent to a hydrogen atom

3. Positive (P): [*+] atom with a positive charge; [#7H2] nitrogen atom of an NH_2 _group

4. Negative (N): [*-] atom with a negative charge; [C&$(C(= O)#8H1), P&$(P(= O)O), S&$(S(= O)O)] carbon, sulfur or phosphorus atom of a COOH, SOOH, or POOH group (SMARTS replaced by a direct graph search)

5. Lipophilic (L): [Cl, Br, I] chlorine, bromine, or iodine atom; [S;D2;$(S(C)(C))] sulfur; atom adjacent to exactly two carbon atoms; sulfur atom adjacent to only carbon atoms (SMARTS replaced by a direct graph search)

### Encodings

#### Topological Fingerprints

All encodings described in the following section rely on the *d *parameter which constrains the maximum topological distance allowed between two atoms *a_i_, a_j _*in a feature.

##### All-Path Encoding (DFS)

All-path encodings are paths generated by a graph traversal with a modified depth-first search as proposed by Ralaivola et al. [6]. The linear fragments are obtained by iterating over all atoms in a molecular graph and performing an exhaustive search up to a predefined depth *d*. To generate an unique representation for each path, a temporary path object is generated and mapped to two alphanumerical string representations by generating the original and reverse string representation of the corresponding path object. The version with the higher lexicographical order is stored.

(4) $$F(C)=\overset{n}{\underset{i}{\cup }}DFS({\hat{a}}_{i},\;d)$$

##### All-Shortest Path Encoding (ASP)

The ASP encoding equals the DFS encoding with the exception that only the paths from an atom are stored that have shortest distances from the root atom to the last atom contained in the path, which leads to a sparser representation. During the depth-first search, all paths are removed from the temporary set of depth-first search paths that do not fulfill this constraint. To incorporate this information the *T_ij _*matrix is required. Let |*p_ij_*| be the length (number of bonds between the *i*th and the *j*th atom in a path) of a path between atoms *i, j*, then the set of features *F *encoding a compound *C *is

(5) $$F(C)=\overset{n}{\underset{i}{\cup }}\left\{DFS({\hat{a}}_{i},\;d),\;|p_{ij}|\;=\;t_{ij}\right\}.$$

Thus, the all-shortest path encoding is a subset of the paths contained in the DFS fingerprint. It is similar to topological atom pair approaches [8] with the exception that all-shortest paths between two atoms are explicitly stored. Borgwardt et al. proposed a graph kernel based on the set of all-shortest paths [22], however, only the vertex pairs and their shortest-path distances were included in this work. The explicit generation of paths is necessary because the Floyd-Warshall algorithm computes only the shortest distances between two vertices.

##### Topological Atom Pairs (AP2D)

This encoding contains atom types and the shortest path distance information between all pairs of atoms. It can be directly extracted from *T_ij _*by converting the pattern ${\hat{a}}_{i}⊕t_{ij}⊕{\hat{a}}_{j}$ where *i *≠ *j *to a string feature. The features are canonicalized by generating the patterns from both reading directions and storing the version with the higher lexicographical order.

(6) $$F(C)=\overset{n}{\underset{i,j}{\cup }}{\hat{a}}_{i}⊕t_{ij}⊕{\hat{a}}_{j}$$

For 2-point patterns this can be easily conducted by regarding the upper half of the distance matrix *T_ij _*only (i.e. *i *>*j*). O(*n*^2^) is needed for the generation of features and O(*n*^3^) for the computation of *T_ij_*.

Thus, the total computation time is cubic.

##### Topological Atom Triplets (AT2D)

This encoding extends the AP2D encoding by a further atom. The set of patterns consists of atom triplets and the topological distance to the next atom in the feature ${\hat{a}}_{i}⊕t_{ij}⊕{\hat{a}}_{j}⊕t_{jk}⊕{\hat{a}}_{k}⊕t_{ki}$ where *i *≠ *j *≠ *k *and *t_ij_, t_jk_, t_ki _*≤ *d*. The total computation time for the features is cubic because all possible combinations of heavy atoms *a*_*i*_, *a*_*j*_, *a*_*k *_have to be considered in the worst case.

(7) $$F(C)=\overset{n}{\underset{i,j,k}{\cup }}{\hat{a}}_{i}⊕t_{ij}⊕{\hat{a}}_{j}⊕t_{jk}⊕{\hat{a}}_{k}⊕t_{ki}$$

##### Topological Autocorrelation Keys (CATS2D)

The CATS2D descriptors encode the pairwise topological relationships of PPP patterns in a molecular graph by a vector of fixed size. The approach was described by Schneider et al. [23], a list of PPP patterns was presented by Renner et al. [7]. The PPPs are defined in the subsection ("Atom Types and Pharmacophore Types"). The combination of all points leads to 15 possible pairs. The pairs are mapped to a key with a fixed dimensionality. The position of a feature in the key is determined by the index for the corresponding pattern pair of PPPs plus the topological distance. For example, this means the bit 76 in the CATS vector with *d *= 9 belongs to the PPP pair DN, and contains the number of pairs with distance 6. In the original publication of the topological CATS descriptors, *d *= 9 was used as the distance cut-off for the topological search, resulting in a vector with 150 dimensions. In our implementation, the search depth can be adjusted by altering the *d *parameter. The resulting vector has (*d *+ 1) · 15 dimensions. The complete list of possible PPP pairs in the vector (block index in parentheses) is: AA (0), AD (1), AL (2), AN (3), AP (4); DD (5); DL (6); DN (7); DP (8); LL (9), LN (10), LP (11), NN (12), NP (13), PP (14). Let *F *(*C*) = *X *be a decomposition into all valid PPP pairs. Then, the CATS2D vector is

(8) $$\vec{x}\in {\mathbb{N}}^{d\cdot 15}\text{ where }x_{\text{offset}(p)\text{+}d_{2D}(p)}=\varphi_{p}(X)$$

where offset(*p*) returns the predefined start index for the pattern *p*, *d*_2*D*_(*p*) returns the topological distance between two atoms in the PPP pair, and *φ_p_*(*X*) counts the number of occurrences of a pattern *p *in *X*.

##### Pharmacophore Pair and Triplet Encodings (PHAP2PT2D, PHAP3PT2D)

The PHAP2PT2D encoding is computed similarly to the AP2D. However, instead of atom types, the information of all PPPs of an atom is used to generate the fingerprint. Thus, we have to iterate over all PPPs of an atom. Analogously, the PHAP3PT2D encoding is computed, which uses three points. To keep the notation simple, let *P_i _*denote the set of valid PPPs for the *i*th atom. Then, the set of valid 2-point pharmacophores is

(9) $$F(C)=\overset{n}{\underset{i,j}{\cup }}P_{i}⊕t_{ij}⊕P_{j}⊕t_{ji}$$

and the set of valid 3-point pharmacophores is defined as

(10) $$F(C)=\overset{n}{\underset{i,j,k}{\cup }}P_{i}⊕t_{ij}⊕P_{j}⊕t_{jk}⊕P_{k}⊕t_{ki}$$

where *t_ij_, t_jk_, t_ki _≤ d*. Actually, there are three additional inner loops over all valid pharmacophore points at atoms *i, j, k*. The complexity of these inner loops is theoretically 5^3 ^because the cardinality of the set of PPPs is 5. However, this complexity is further reduced because the meaning of some PPP definitions is contradicting for some combinations, such as "atom is positively charged" and "atom is negatively charged". The overall complexity is *O*(*n*^3^) because of the constant computation time of the inner loops.

##### SHED Key (SHED)

The SHED Keys are closely related to the pharmacophore atom pair based encodings, with the following major differences: First, the number of dimensions is fixed, second the entries do not describe a count but the entropy of the respective atom pair descriptor [24]. The implementation differs slightly from the original implementation because it utilizes the PPP definitions as described by Renner et al. [7]. The distribution is analyzed for all possible combinations of PPPs. From that distribution of pairwise features the Shannon entropy is calculated as the descriptor in the corresponding PPP pair dimension of the SHED Key. The Shannon entropy of a PPP pair PPP*_i _*is defined as

(11) $$\vec{x}\text{ where }x_{i}=H({\text{PPP}}_{i})=-\sum_{l=1}^{d}p{\text{(PPP}}_{i}\text{(}l\text{)) }\log_{b}p({\text{PPP}}_{i}(l))$$

where PPP*_i_*(*l*) denotes the *i*th PPP pair that is separated by a topological distance *l*. If pattern *i *was not found, the value of the *i*th dimension is set to 0. The distribution of a PPP pair is calculated by regarding the different distances 1, 2, ...,*l*, ..., d. The resulting vector has 15 real-valued entries.

##### Extended Connectivity Fingerprints (ECFP)

We implemented a variant of the ECFP as described by Rogers and Hahn [5]. Each ECFP feature represents a circular substructure around a center atom. The algorithm starts with the initial atom identifier of the center atom and grows a circular substructure around this atom throughout a defined number of iterations (search depth). For each round, the current extended version of the feature is added to the final set of features. In contrast to other radial fingerprints, the bonding information is included. Therefore, a feature can be extracted, for example, as canonical SMILES.

The current implementation of the ECFP in jCompoundMapper differs slightly from the original implementation. In the original algorithm, the identifiers of the alpha atoms of a center atom are used to calculate an updated identifier for the center atom. The algorithm only includes the alpha atoms of a center atom in each iteration and thus the connectivity information is completely discarded between the layers. However, the identifier of a center atom implicitly contains information from further and further away of the center atom in each iteration because the atom identifiers of the previous iteration are used. We explicitly model the growing substructure by using the initial atom identifiers in each iteration and keeping the connectivity information between the layers. After an iteration, new possible attachment points for a specific circular substructure are kept in memory and those attachment points are extended in the next iteration.

##### Topological Molprint-like fingerprints (RAD2D)

This encoding was proposed by Bender et al. [4,25] and describes the radial environment by the atoms with the topological distance 1, 2, ..., *l*, ..., *d *rather than the full paths containing bonds. A shell *s*(*a_i_*)*_l _*in our implementation contains the canonically sorted set of topological neighbors of atom *a_i _*at a distance *t_ij _*= *l*. Additionally, we include the concatenation of all shells ≤ 1, 2, ..., *l*, ..., d as additional features. Therefore, a resulting set of features contains *n · d *features.

(12) $$F(C)=\overset{n}{\underset{i}{\cup }}\underset{d}{\cup }\;can\text{ (}s{\left({\hat{a}}_{i}\right)}_{d}\text{)}$$

##### Local Path Environments (LSTAR)

This fingerprint is a radial fingerprint similar to RAD2D. The major difference is that all paths up to depth *d *are stored in a shell. First, the tree of all paths originating from an atom *a_i _*is generated. Then, all paths of a certain length are assigned to a shell *s*(*a_i_*)*_d _*containing the paths originating from root atom *a_i _*of length *d*. This is equal to a canonical representation of *DFS*(*a*_*i*, _*d*) in a single canonical feature. The paths in a shell are sorted in lexicographical order to be comparable. The resulting fingerprint contains all shells ≤ 1, 2, ..., *l*, ..., *d*. The major difference to the Molprint-like fingerprints is that the bond information is still included.

(13) $$F(C)=\overset{n}{\underset{i}{\cup }}\underset{d}{\cup }can\text{ (}DFS({\hat{a}}_{i},\;d)\text{)}$$

#### Geometrical Fingerprints

All geometrical encodings support the *d *parameter which defines the distance cut-off between two atoms. Another important parameter is the scaling factor *s*, as described at the beginning of this section.

##### Geometrical Atom Pairs and Atom Triplets (AP3D, AT3D)

These encodings are implemented similarly as their topological pendants AP2D and AT2D. The only difference is that *G_ij _*is used for the distance information. Thus, the geometrical two-point atom pair encoding (AP2D) is defined as

(14) $$F(C)=\overset{n}{\underset{i,j}{\cup }}{\hat{a}}_{i}⊕g_{ij}⊕{\hat{a}}_{j}$$

where *i *≠ *j *and *g_ij _*≤ *d*.

For the three-point relationships AT3D, we have

(15) $$F(C)=\overset{n}{\underset{i,j,k}{\cup }}{\hat{a}}_{i}⊕g_{ij}⊕{\hat{a}}_{j}⊕g_{ik}⊕{\hat{a}}_{k}⊕g_{ki}$$

where *i *≠ *j *≠ *k *and *g_ij_, g_jk_, g_ki _*≤ *d*.

This is a standard encoding implemented in several toolkits; a kernel based on such patterns was published by Mahé et al [9].

##### Geometrical CATS fingerprints (CATS3D)

Our implementation differs from the description of the original CATS3D [7], which uses the Molecular Operating Environment (MOE, Chemical Computing Group, http://www.chemcomp.com/) patterns to depict surface features of a molecule. The version implemented in jCompoundMapper uses the PPP definitions which were also used in the implementation of the CATS2D vector. Again, let *F*(*C*) = *X *be the set of all valid PPP pairs and *φ_p_*(*X*) a function which counts a pattern *p *∈ *X*. Then the CATS3D vector is

(16) $$\vec{x}\in {\mathbb{N}}^{d\cdot 15}\text{ where }x_{\text{offset(}p\text{)}+d_{3D}(p)}=\varphi_{p}(X)$$

where *d*_3*D*_(*p*) returns the geometrical distance of the two atoms, which equals *g_ij _*between any atoms *i*, *j *contained in a feature.

##### Geometrical pharmacophore fingerprints (PHAP2PT3 D, PHAP3PT3D)

These fingerprints are derived from their topological variants PHAP2PT2D and PHAP3PT2D by replacing the *T_ij _*matrix by *G_ij_*. Let *P_i _*denote the set of valid PPP for the *i*th atom then *P_i _*⊕ *g_ij _*⊕ *P_j _*is a valid two-point pharmacophores and *P_i _*⊕ *g_ij _*⊕ *P_j _*⊕ *g_jk _*⊕ *P_k _*⊕ *g_ki _*is a valid three-point pharmacophore, where *g_ij_*, *g_jk_*, *g_ki _*≤ *d*.

The set of valid 2-point pharmacophores is defined as

(17) $$F(C)=\overset{n}{\underset{i,j}{\cup }}P_{i}⊕g_{ij}⊕P_{j}$$

and the set of valid 3-point pharmacophores is defined as

(18) $$F(C)=\overset{n}{\underset{i,j,k}{\cup }}P_{i}⊕g_{ij}⊕P_{j}⊕g_{ik}⊕P_{k}⊕g_{ki}.$$

Again, *P_i _*denotes the set of valid PPPs for the *i*th atom.

##### Geometrical Molprint-like fingerprints (RAD3D)

These encodings are the geometrical pendant of the topological RAD2D encoding. Similar to the RAD2D encoding, the atoms with *g_ij _*= *l *at a binned geometrical distance are added to a shell descriptor. For each value in 1, 2, ..., *l*, ..., *d *a pattern containing all shells up to distance *l *in a canonical order is created. Therefore, the encoding contains *n *· *d *entries.

(19) $$F(C)=\overset{n}{\underset{i}{\cup }}\underset{d}{\cup }can\text{ (}s{\left({\hat{a}}_{i}\right)}_{d}\text{)}$$

#### Example of encodings

**Figure 1:**
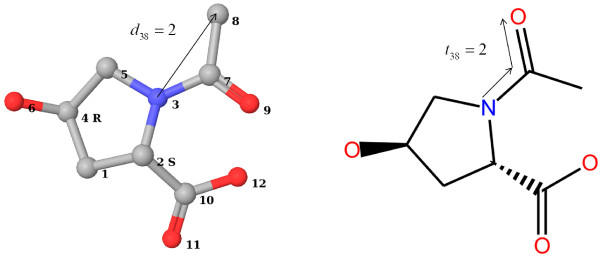
**Topology and Geometry of Oxaceprol**. The geometry and topology of Oxaceprol. Pharmacophore types shown in the 3 D structure are 1 = [L], **3 = [A - [#7H0]]**, 6 = [D - [OH], A - [O]],8 = [L], 9 = [A - [O]],10 = [N], 11 = [A - [O]], 12 = [D - [OH], A - [O]]. The geometry and topology of this compound is the basis for the exemplary fingerprints shown in Table 1. Note that multiple PPPs can be assigned to an atom: In this case atoms 6 and 12 have two valid PPPs.

Comparison Table 1 gives a direct tabular comparison of the features extracted by the encodings together with their count or value. The features are generated from the compound presented in Figure 1.

**Table 1:** Examples of Encodings

Encoding	Eq.*^a^*	c param*^b^*	Pattern produced by *f*
DFS	4	0	C.2-N.3-C.3:1, C.3-C.2-N.3:1, C.3-N.3-C.3:1, C.3-N.3:1, C.2-C.3-N.3:1, C.3-C.3-N.3:1, ...
ASP	5	1	N.3-C.3 = O.1:1, C.1-C.3-N.3:1, C.2-N.3:1, C.2-N.3-C.3:1, C.3-C.2-N.3:1, C.3-N.3:1, ...
AP2D	6	2	N.3-1-C.2:1, N.3-1-C.3:1, N.3-2-C.2:1, N.3-2-C.1:1, N.3-2-C.3:1, O.1-2-N.3:1
AT2D	7	3	C.2-2-N.3-1-C.3-1:1, N.3-2-C.2-2-C.2-1:1, N.3-2-C.2-2-C.3-2:1, ...
CATS2D	8	6	0:5, 2:2, 3:4, ...
PHAP2POINT2D	9	8	A-2-A:1, L-2-A:1, N-2-A:1
PHAP3POINT2D	10	9	A-2-A-2-L-2:1, A-2-A-2-L-2:1, ...
SHED	11	14	AA:2.8, AL:3.596, AN:2.872, ...
ECFP	-	12	[*]N([*])C(= O)C:1, [*] = C([*])N(C[*])C([*])[*]:1, [*]N([*])[*]:1, ...
RAD2D	12	15	0[N]1[C C C]:1, 0[N]1[C C C]2[C C C C O]:1
LSTAR	13	13	[N.3-C.2, N.3-C.3, N.3-C.3]:1, [N.3-C.2-C.3, N.3-C.3-C.1, N.3-C.3-C.2, N.3-C.3-C.3, N.3-C.3 = O.1]:1

AP3D	14	4	N.3-1-C.2:1, N.3-1-C.3:1, N.3-2-C.1:1, N.3-2-C.2:1, N.3-2-C.3:1, O.1-2-N.3:1
AT3D	15	5	C.3-1-O.1-2-N.3-1:1, O.1-2-C.1-2-N.3-2:1, C.2-2-C.2-2-N.3-1:1, ...
CATS3D	16	7	0:5, 2:2, 3:4, ...
PHAP2POINT3D	17	10	A-2-A:1, L-2-A:1, N-2-A:1
PHAP3POINT3D	18	11	A-2-A-2-L-2:1, A-2-L-2-A-2:1, L-2-A-2-A-2:1
RAD3D	19	16	0[N.3]1[C.2 C.3 C.3]2[C.1 C.2 C.3 C.3 O.1]:1, 0[N.3]1[C.2 C.3 C.3]:1

Examples and numeric values (counts, or, in the case of SHED, entropies) for the string representations for the different encodings of Oxaceprol (Figure 1) with *d *= 2, default element atom typing and delimiter symbol "-". The patterns are extracted around the nitrogen atom. For some of the encodings, only a subset of the features is shown.

^*a *^Equation number *^b ^*Parameter for command-line interface

## Implementation

### Third-party libraries

The underlying chemical expert system is the Chemistry Development Kit (CDK) [3,26] in its current development version 1.35. It provides the basic functionality for parsing, typing, and graph algorithms for molecular data. For the command-line interface we employed the Apache Commons command-line parser 1.2 http://commons.apache.org/cli/. The access via the API or the binary using the command-line interface enables the user to utilize the library for batch processing. The language level is Java 1.6.

### Additional functionality

#### Import and Export of Data

The valid input format is MDL SD format with attached hydrogens for the command-line tool. The CDK molecule objects can be processed using the API.

There are exporters for various formats of popular machine learning toolboxes. The ARFF format is the native WEKA [13] format, the support vector machine libraries LIBSVM and LIBLINEAR are supported by their sparse hashed format and precomputed matrix format. Alternatively, there are several comma-separated formats which support hashed or string features, which can be imported into toolboxes like R or MATLAB.

jCompoundMapper includes a buffered random access reader for parsing the input files. Thus, it can read files of the maximum size supported by the Java runtime environment. The memory requirements are low if the encodings are exported sequentially (such as the sparse LIBSVM format) because only a single encoding has to be stored at a time. If the computation of a similarity matrix is required, all encodings are kept in memory to ensure a fast computation of similarities. For this reason, a matrix computation requires additional memory for large data sets.

The label or class for learning tasks is read from the SD property and is integrated into the specific output format. As for the ARFF format, a nominal or numeric class label is created, depending on the distribution of labels in the input format. The user may overwrite the default threshold for the number of classes (currently, this is set to five).

#### Hashing

All decomposition algorithms *F*(*C*) return the full list of features (the encoding). A feature *f *has an integer identifier *f*.*id *∈ {0, 1, .... 2^32^} which allows the efficient use in hash based collections. Therefore, it is possible to operate on the full set of descriptors and to generate hashed fingerprints. Hashing is useful to generate binary vectors of a fixed size. The hash function *H*

$$H\circ F(C)\to {\mathbb{Z}}_{2}^{h}$$

is used to project the set of features to a binary vector of the dimension *h*. The size depends on the expected number of features (see Table 1). *H *generates the hashed bit of a pattern depending on the numerical seed *f.id *assigned to each feature. In most cases, the seed equals a hash code of the string representation. The hashing step is also useful to obtain nominal features for fast comparisons. A nominal feature is a feature *f *with a finite set of possible values like *f *∈ {red, green, blue} or, convenient for chemical graphs, *f *∈ {pattern included, pattern not included}.

#### Similarity Matrices

jCompoundMapper offers a FeatureMap data structure, which stores the features in a wrapped hash map. Different metrics are defined on this data structure, such as MinMax and Tanimoto [27]. Thus, it is possible to compute distance matrices within seconds on an average desktop computer. Now, we assume two mappings *F *(*C_A_*) = *A *and *F *(*C_B_*) = *B*. Further, let *φ_p_*(X) count the number of occurrences of pattern *p *∈ *X*.

The MinMax similarity is defined as

$$\text{MinMax(}A,B\text{)}:=\frac{\sum_{p\in X}\min(\varphi p(A),\varphi p(B))}{\sum_{p\in X}\min\left(\varphi p(A),\varphi p(B)\right)}.$$

The Tanimoto similarity can be used instead if only the occurrence of a pattern is taken into account

$$\text{Tanimoto}\;(A,B):=\frac{\left|A\cap B\right|}{\left|A\cup B\right|}.$$

The feature maps permit to compute similarity matrices with jCompoundMapper on the full set of features of a compound *C*, without introducing noise by hashing. Nevertheless, it is also possible to generate hashed binary fingerprint objects of any of the encodings.

## Results and Discussion

### Computation Times Benchmarks

Table 2 presents the performance of the different encodings as implemented in jCompoundMapper. The computation time for the atom-based approaches varies from the atom pair encodings which can be computed with 332-339 molecules per second to the depth- first search based encodings which have a performance of 68-136 molecules per second. The encodings relying on the typing using the PPP SMARTS definitions are significantly slower with about 7-8 processed molecules per second. The conversion time includes reading, typing, and feature map creation. As benchmark data set, we chose the publicly available ChemDB random background data set published in the virtual screening study by Nasr et al. [28]. This data set comprises 175,000 random compounds from ChemDB [29].

**Table 2:** Conversion Time

Encoding	param*^b^*	molecules/s	mean f.*^a^*	max f.*^a^*	median f.*^a^*	complex. Mem.*^c^*
DFS	d = 8, a = EN	68.6	396	3,554	362	O(*nα^d^*)
ASP	d = 8, a = EN	112.1	216	1,198	204	O(*nα^d^*)
AP2D	d = 8, a = EN	339.8	95	256	96	O(*n*^2^)
AT2D	d = 5, a = EN	91.6	1,848	7,922	1,811	O(*n*^3^)
CATS2D	d = 9, a = PPP	6.9	150	150	150	O(1)
PHAP2PT2D	d = 8, a = PPP	6.8	35	132	34	O(*p*^2^)
PHAP3PT2D	d = 5, a = PPP	6.7	300	1,664	277	O(*p*^3^)
SHED	d = 8, a = PPP	8.0	15	15	15	O(1)
ECFP	d = 4, a = DIR	181.0	77	349	77	O(*nα^d^*)
RAD2D	d = 3, a = EN	232.5	55	192	55	O(*nd*)
LSTAR	d = 6, a = EN	136.6	144	884	143	O(*nd*)

AP3D	d = 10, a = EN	332.5	112	336	113	O(*n*^2^)
AT3D	d = 6, a = EN	71.4	3,450	27,774	3,188	O(*n*^3^)
CATS3D	d = 9, a = PPP	6.6	150	150	150	O(1)
PHAP2PT3D	d = 10, a = PPP	7.2	41	189	40	O(*p*^2^)
PHAP3PT3D	d = 6, a = PPP	7.1	660	5,844	601	O(*p*^3^)
RAD3D	d = 4, a = EN	227.1	85	611	85	O(*nd*)

Computation time on a random ChemDB [29] background data set consisting of about 175,000 compounds. This includes reading, typing, and generation of the feature map objects. Empirical computation time was obtained on a dual core AMD Opteron(tm) Processor 280, 2.4 GHz, 2 GByte RAM, Java Runtime Environment 1.6, Linux kernel 2.6.18, using a single core.

*^a^*unique features, EN: element neighbor, PPP: possible pharmacophore points, DIR: Daylight Invariants plus ring flag *^b ^*standard parameters of the implemented encodings *^c ^d*: search depth, *p*: number of possible pharmacophore points

### Machine Learning Performance

A major application of molecular encodings are structure-based machine learning and data mining methods. The aim of such applications is either the prediction of molecular properties or the ranking of compounds according to a trained model. In the following experiments, we wanted to assess the quality of the encodings implemented in jCompoundMapper for several established regression and classification benchmark problems. The encodings were used with the default parameters as given in Table 2. The compounds were prepared using CORINA [30] for initial coordinates and were refined using Schrödinger MacroModel [31] with the OPLS 2005 force field.

#### QSAR Regression Problems with LIBSVM

LIBSVM [12] is a library for support vector machines. For the experiments on the regression benchmarks, we decided to train *ϵ*-support vector regression on the benchmark compilation of eight pIC50 QSAR problems published by Sutherland et al. [14]. The Gram matrices were precomputed by the MinMax similarity, which is also a valid kernel function. We conducted these experiments to find out whether there are significant differences between the performances of the different encodings.

We evaluated the nested cross-validation performance of the different encodings and compared the outcome of the experiments against results from the literature. The parameters *C *and *ϵ *of the support vector machine were selected in a nested cross-validation. In the experiments, a 10-fold cross-validation was repeated 20 times using an initial seed value. Therefore, the values represent the mean and the standard deviation, computed over 200 models. Based on these statistics, the corrected resampled *t*-test of Bouckaert and Frank [32] can be applied. The results are summarized in Table 3. With the exception of THERM and THR, the performance of the best encodings was at least comparable to the mean squared error values for a sophisticated graph kernel given by Fechner et al. [33] on the same benchmarks.

**Table 3:** Nested Cross-validation MSE Performance on the Sutherland Data Sets

Encoding	ACE	ACHE	BZR	COX2	DHFR	GPB	THERM	THR
DFS	1.73 ± 0:74	0.66 ± 0.28	0.61 ± 0.31	1.13 ± 0.30	**0.57 **± **0.21**	0.66 ± 0.54	2.10 ± 1.31	0.60 ± 0.38
ASP	1.70 ± 0.72	**0.62 **± **0.26**	0.53 ± 0.27	1.11 ± 0.30	**0.58 **± **0.21**	0.63 ± 0.48	2.09 ± 1.32	0.59 ± 0.38
AP2D	**1**.**50 **± **0**.**70**	0.85 ± 0.37	0.70 ± 0.37	1.03 ± 0.30	0.73 ± 0.29	0.61 ± 0.45	2.19 ± 1.20	0.50 ± 0.31
AT2D	1.57 ± 0.69	0.74 ± 0.34	0.69 ± 0.35	0.97 ± 0.27	0.66 ± 0.30	0.60 ± 0.47	1.97 ± 1.20	**0.49 **± **0.32**
CATS2D	1.76 ± 0.72	0.93 ± 0.33	0.89 ± 0.45	1.35 ± 0.43	0.69 ± 0.19	0.64 ± 0.45	2.28 ± 1.14	0.52 ± 0.32
PHAP2PT2D	1.77 ± 0.71	0.96 ± 0.33	0.91 ± 0.45	1.38 ± 0.44	0.72 ± 0.20	0.65 ± 0.48	2.18 ± 1.10	0.53 ± 0.31
PHAP3PT2D	1.81 ± 0.69	0.96 ± 0.33	0.82 ± 0.39	1.23 ± 0.41	0.67 ± 0.21	**0.56 **± **0.49**	**1.89 **± **1.16**	0.57 ± 0.37
SHED	2.08 ± 0.76	1.05 ± 0.50	1.09 ± 0.46	1.64 ± 0.48	1.49 ± 0.35	0.70 ± 0.33	2.71 ± 1.54	0.49 ± 0.28
ECFP	1.80 ± 0.77	0.72 ± 0.29	0.66 ± 0.32	1.01 ± 0.28	**0.57 **± **0.20**	0.68 ± 0.55	2.19 ± 1.36	0.51 ± 0.33
RAD2D	1.87 ± 0.75	0.77 ± 0.33	0.79 ± 0.37	1.08 ± 0.30	0.71 ± 0.27	0.72 ± 0.59	2.20 ± 1.33	0.50 ± 0.35
LSTAR	1.97 ± 0.79	0.72 ± 0.29	0.69 ± 0.30	1.04 ± 0.27	0.62 ± 0.19	0.76 ± 0.61	2.31 ± 1.39	0.50 ± 0.31

AP3D	1.60 ± 0.69	0.69 ± 0.32	0.59 ± 0.32	**0.93 **± **0.27**	0.67 ± 0.23	0.67 ± 0.51	2.73 ± 1.35	0.57 ± 0.33
AT3D	1.77 ± 0.68	0.64 ± 0.28	0.67 ± 0.36	0.99 ± 0.28	0.57 ± 0.18	0.74 ± 0.60	2.75 ± 1.37	0.60 ± 0.28
CATS3D	1.75 ± 0.70	0.90 ± 0.38	0.81 ± 0.36	1.31 ± 0.41	0.73 ± 0.20	0.79 ± 0.49	2.47 ± 1.32	0.62 ± 0.32
PHAP2PT3D	1.75 ± 0.70	0.87 ± 0.36	0.81 ± 0.40	1.32 ± 0.41	0.73 ± 0.20	0.83 ± 0.54	2.53 ± 1.30	0.65 ± 0.33
PHAP3PT3D	1.99 ± 0.77	0.82 ± 0.29	0.81 ± 0.36	1.14 ± 0.37	0.59 ± 0.17	0.86 ± 0.69	2.84 ± 1.46	0.69 ± 0.30
RAD3D	2.17 ± 0.78	0.78 ± 0.31	0.73 ± 0.35	1.10 ± 0.30	**0.57 **± **0.17**	0.82 ± 0.69	2.79 ± 1.37	0.58 ± 0.31

Overview of *ϵ *support vector MSE regression performance on the Sutherland data set using the MinMax kernel function. The performance was evaluated using a nested 10-fold cross-validation repeated 20 times. Bold values indicate the best encodings according to the number of signicant better comparisons (*p *≤ 0.05) against the other encodings. The *p*-values were determined by the corrected resampled *t*-test [32].

An analogue setup was used to compute nested leave-one-out cross-validation results to compare the predictive performance of the support vector machine in combination with the jCompoundMapper encodings to literature results. Again, we optimized the parameters *C *and *ϵ *(we used a 10-fold cross-validation repeated 2 times to select the best parameter combination in the inner loop) and trained a model for each of the *n *leave-one-out sets and predicted the external sample for each model.

Table 4 and 5 summarize the results of the nested leave-one out cross-validation according to the mean squared error and Pearson's correlation coefficient. Sutherland et al. used several descriptor-based approaches to model the activity of the benchmark set (presented in Table 4 and 5) using partial least squares (PLS) [14]. Compared against the results (squared correlation coefficient) given for the best descriptor approach presented in this study, the jCompoundMapper encodings are competitive. The findings are summarized in Table 6 which shows a similar performance in two cases, a better performance in three cases, and a worse performance in three cases.

**Table 4:** Nested Leave-one-out MSE Performance on Sutherland Data Sets

Encoding	ACE	ACHE	BZR	COX2	DHFR	GPB	THERM	THR
DFS	1.93	**0.61**	0.61	1.09	**0.57**	0.66	**2.08**	0.55
ASP	1.93	**0.56**	**0.54**	1.07	**0.56**	**0.63**	**2.08**	0.53
AP2D	**1.59**	0.79	0.68	1.04	0.72	0.64	**2.06**	**0.45**
AT2D	**1.68**	0.69	0.67	**0.92**	0.69	0.65	**1.92**	**0.45**
CATS2D	1.83	0.83	0.96	1.34	0.65	**0.62**	2.20	**0.45**
PHAP2PT2D	1.88	0.92	0.98	1.37	0.67	**0.62**	**2.10**	**0.46**
PHAP3PT2D	1.83	0.91	0.85	1.20	0.66	**0.58**	**2.04**	0.50
SHED	2.11	1.00	1.13	1.71	1.41	0.72	2.94	**0.43**
ECFP	2.01	0.66	0.66	**0.96**	**0.57**	0.65	2.17	**0.47**
RAD2D	1.99	0.73	0.79	1.03	0.72	0.67	2.18	**0.43**
LSTAR	2.29	0.66	0.68	1.00	0.60	0.71	2.31	**0.46**

AP3D	1.88	0.64	**0.59**	**0.90**	0.67	0.70	2.61	0.54
AT3D	2.04	0.60	0.65	**0.97**	**0.58**	0.71	2.70	0.59
CATS3D	1.91	0.85	0.84	1.26	0.70	0.74	2.62	0.58
PHAP2PT3D	1.92	0.81	0.85	1.30	0.70	0.76	2.72	0.62
PHAP3PT3D	2.40	0.73	0.81	1.11	**0.59**	0.82	2.82	0.67
RAD3	2.43	0.73	0.73	1.04	0.57	0.75	2.75	0.55

Overview of *ϵ *support vector regression performance on the Sutherland data set using the MinMax kernel function. The performance was evaluated using a nested leave-one-out cross-validation with a parameter optimization using 10-fold cross-validation repeated 2 times. Bold values indicate performances not worse than 10% of the best performing encoding.

**Table 5:** Nested Leave-one-out Correlation Performance on Sutherland Data Sets

Encoding	ACE	ACHE	BZR	COX2	DHFR	GPB	THERM	THR
DFS	**0.79**	**0.78**	**0.71**	0.68	**0.86**	0.69	**0.70**	0.68
ASP	**0.79**	**0.80**	**0.75**	0.69	**0.87**	**0.71**	**0.70**	0.69
AP2D	**0.83**	0.70	0.67	**0.71**	0.82	**0.71**	**0.70**	**0.75**
AT2D	**0.82**	0.74	0.67	**0.74**	**0.83**	**0.70**	**0.73**	**0.75**
CATS2D	**0.81**	0.68	0.49	0.60	0.84	**0.72**	0.68	**0.75**
PHAP2PT2D	**0.80**	0.64	0.48	0.59	**0.83**	**0.72**	**0.70**	**0.74**
PHAP3PT2D	**0.80**	0.64	0.56	0.65	**0.84**	**0.73**	**0.71**	0.71
SHED	0.78	0.61	0.34	0.46	0.61	0.66	0.57	**0.76**
ECFP	0.78	**0.76**	0.68	**0.73**	**0.86**	0.70	0.68	**0.74**
RAD2D	0.78	0.73	0.59	**0.70**	0.82	0.68	0.68	**0.76**
LSTAR	0.75	**0.77**	0.68	**0.72**	**0.86**	0.66	0.66	**0.74**

AP3D	**0.80**	**0.77**	**0.72**	**0.74**	**0.83**	0.67	0.60	0.68
AT3D	0.78	**0.80**	0.68	**0.72**	**0.86**	0.66	0.58	0.65
CATS3D	**0.79**	0.67	0.57	0.63	0.83	0.64	0.60	0.66
PHAP2PT3D	**0.79**	0.69	0.56	0.62	**0.83**	0.63	0.58	0.62
PHAP3PT3D	0.73	0.73	0.58	0.68	**0.86**	0.59	0.55	0.58
RAD3	0.73	0.73	0.63	**0.70**	**0.86**	0.63	0.57	0.69

Overview of *ϵ *support vector regression Pearson's correlation coefficient performance on the Sutherland data set using the MinMax kernel function. The performance was evaluated using a nested leave-one-out cross-validation with a parameter optimization using 10-fold cross-validation repeated 2 times. Bold values indicate performances not worse than 5% of the best performing encoding.

**Table 6:** Comparison with Literature Results on Sutherland Data Sets

Data set	Best Encoding	$Q_{loo}^{2}$	Sutherland	$Q_{loo}^{2}$
ACE	AP2D	0.69	HQSAR	0.72
ACHE	ASP, AT3D	**0.57**	CoMSIA	0.49
BZR	ASP	**0.56**	CoMSIA	0.45
COX2	AT2D	0.55	CoMSIA	0.57
DHFR	ASP	**0.76**	CoMSIA	0.69
GPB	PHAP3PT2D	0.53	HQSAR	**0.66**
THERM	AT3D	0.53	2.5D	**0.66**
THR	RAD2 D, RAD3D	0.58	CoMSIA	**0.72**

Comparison to the best squared correlation coefficients given by Sutherland et al. (PLS on various descriptors), bold font indicate a result which is at least 5% better.

#### Classification of Toxic Compounds with LIBLINEAR

Another increasingly important task is to build models on large data sets of chemicals. A machine that can cope with such a setup is LIBLINEAR [16], a linear large-scale support vector machine. The large Ames data set was published by Hansen et al. [15] and contains 6512 compounds and their measured toxicity in an Ames test. We skipped the encodings based on the PPP typer because several compounds do not have any pharmacophore point according to the PPP definition. The results were obtained by tuning the *C *parameter in log_2 _∈ {-8, -7, ..., 2} within a 2-fold cross-validation on the training set and evaluating the model performance on five defined splits as described in [15].

Table 7 shows the AUC ROC results for the large Ames toxicity benchmark set. The ECFP and LSTAR encodings achieved the best results, comparable to the results of several supervised classifiers presented by Hansen et al. [15] using dragonX [34] descriptors. Hansen et al. applied *k*-nearest neighbor, support vector machines with a radial basis function, Gaussian processes, and random decision forests to build models on dragonX descriptors for this problem. The approaches were evaluated on the same defined splits. The performance of LIBLINEAR with the best encodings is comparable to the best approaches Gaussian processes and support vector machines. Even the worst performing encodings (AP2D, AP3D, and DFS) were competitive to the *k*-nearest neighbor classifier on dragonX descriptors.

**Table 7:** Comparison with Literature Results on the Ames Toxicity Benchmark

Encoding	AUC ROC*^a^*
ECFP	0.87 ± 0.01
LSTAR	0.86 ± 0.01
SVM-dragonX*^b^*	0:86 ± 0.01
RAD2D	0.85 ± 0.02
RAD3D	0.85 ± 0.01
ASP	0.85 ± 0.02
GP-dragonX*^b^*	0.84 ± 0.01
AT2D	0.83 ± 0.01
RF-dragonX*^b^*	0.83 ± 0.01
AT3D	0.81 ± 0.01
*k*NN-dragonX*^b^*	0.79 ± 0.01
DFS	0.78 ± 0.01
AP2D	0.78 ± 0.03
AP3D	0.76 ± 0.02

Benchmarks for the large Ames toxicity benchmark using LIBLINEAR (nested 5-fold cross-validation on defined splits). The features were hashed to 2^14 ^bit sparse binary fingerprints.

*^a ^*Area under the ROC curve *^b ^*Reference classifier from the study of Hansen et al. using dragonX 1.2, SVM: support vector machine, GP: Gaussian processes, RF: random decision forest, *k*NN: *k*-nearest neighbor

### Java API and Command-line Interface

#### Java API Usage Example

The core of the library is a Java API. The API enables to process chemical information from an abstract level, similar to a workflow tool. The example given in Appendix 1 reads an MDL SD file, converts the compounds to feature maps and calculates all pairwise similarities.

#### Command-Line Interface Example

**Figure 2:**
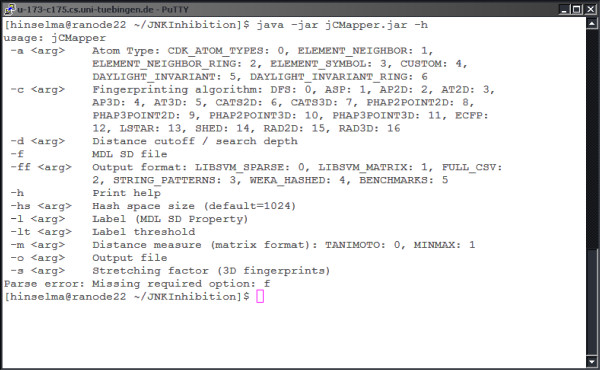
**Command-line Interface**. The binary can be accessed via a command-line interface, which allows for scripting.

The following section gives an example of using the binary executable version of jCompoundMapper. As a case in point, this version can be used in shell scripts. Calling the command-line tool using -h gives an overview of possible parameters (see Figure 2).

Using the defaults (or via -ff 0), jCompoundMapper generates a hashed LIBSVM output format using the depth-first search encoding with element plus neighbor count atom types.

In the following, we process the training and the known test set from the environmental toxicity challenge http://www.cadaster.eu/node/65) which were converted to MDL SD format. The label (MDL property) to be learned is log(IGC50-1). Using these settings, the structures of the training set were mapped to hashed fingerprints with the default settings.

java -jar jCMapperCLI.jar -f challenge_train.sdf -l "log(IGC50-1)"

After the computation, an overall statistic is plotted showing e.g. the average number of features in the fingerprints. In the next step, we map the test file to the same representation. Bits in the test file were set in exactly the same positions in the vector because the random numbers are generated by using the seed value defined by the features.

java -jar jCMapperCLI.jar -f challenge_test_known.sdf -l "log(IGC50-1)"

In the next step, a cross-validation is conducted by using the precompiled binary distribution of LIBSVM that can be downloaded from the LIBSVM homepage. The parameters are set as follows: -t 0 sets the linear kernel (dot product), -s 3 sets ∈ regression, and -c 2 sets the error weight to 2. The file for training was produced in the previous step.

svmtrain -t 0 -s 3 -v 10 -c 2 challenge_train.DFS.LIBSVM_SPARSE

LIBSVM produces no model in cross-validation mode. However, the LIBSVM cross-validations statistics showed that the model has an *MSE *of 0.32 and an *Q*^2 ^of 0.71, indicating a reasonable parametrization.

Cross Validation Mean squared error = 0.324891

Cross Validation Squared correlation coefficient = 0.712412

Finally, the model is trained by omitting the cross-validation flag -v.

svmtrain -t 0 -s 3 -c 2 challenge_train.DFS.LIBSVM_SPARSE

This step produces a separate model file, which can be used to predict the external test set that was generated during the second step. This is conducted by calling svmpredict.

svmpredict challenge_test_known.DFS.LIBSVM_SPARSE challenge_train.DFS.LIBSVM_SPARSE.model result

The results are printed by LIBSVM highlighting that the performance on the external test set is *MSE *= 0.29 and *R*^2 ^= 0.74. The result on the known test of the environmental toxicity prediction challenge would be in the top ranks of the competition. The prediction values can be obtained by opening the LIBSVM output file result in an editor.

Mean squared error = 0.291011 (regression)

Squared correlation coefficient = 0.742283 (regression)

## Conclusions

jCompoundMapper is an open source library for molecular fingerprinting with a focus on machine learning and data mining applications. A command-line interface exists for the user who is not familiar with programming, which allows a simple usage from the shell or the application in scripts. The architecture provides the functionality to derive fingerprints from existing ones or to integrate own encodings. In contrast to closed source fingerprinting toolkits, a scientist knows exactly how the fingerprint is computed (like the labeling function, distance cut-offs) and can even inspect the source code of the generation routine. We compared the performance using linear and non-linear support vector machines on standard machine learning benchmarks in the research field. The results show that the machine learning performance using the encodings with default parameters is already close to more sophisticated state-of-the-art descriptors. The binary version provides a command-line interface allowing for the generation of models from the shell with open source software such as LIBSVM or WEKA in reasonable time on average desktop computers. The library itself uses only functionality of open source software licensed under the LGPL. Therefore, the library can be used in any project compatible with the CDK. Further projects with the library, such as a KNIME node wrapping jCompoundMapper, are planned.

## Availability

The following files are available for download from http://jcompoundmapper.sourceforge.net/

1. External library, which can be integrated as Java jar library file

2. External library, including sources

3. Binary command-line tool (requires a Java runtime environment) and a short tutorial with a prepared data set

## Competing interests

The authors declare that they have no competing interests.

## Authors' contributions

GH wrote most of the code and the manuscript. LR implemented the Molprint-like fingerprints and the extended connectivity fingerprint, helped to design the library, and participated in writing the manuscript. AJ implemented an initial version of the pharmacophore typer and the CATS2D descriptors. NF tested some of the encodings in experiments and helped to develop the atom typing schemes. AZ supervised the study and participated in the discussion of the results. All authors read and approved the final manuscript.

## Appendix

### Appendix 1 - Usage of the API

Example of using the API: Read molecules, map the compounds to encodings, and compute a similarity matrix.

1 // read sd file

2 RandomAccessMDLReader reader = **new **RandomAccessMDLReader(**new **File (" molecules . sdf " ) );

3

4 // convert all compounds in the data set to feature maps

5 final ArrayList <FeatureMap> featureMaps = **new **ArrayList <FeatureMap >( );

6 final FingerPrinter finger printer = **new **Encoding2DAllShortestPaths ( ) ;

7 **for **(int i = 0; i < reader. getSize ( ); i++) {

8    ArrayList <IFeature > rawFeatures = finger printer . getFingerprint (reader. getMol (i));

9    featureMaps . add (**new **FeatureMap (rawFeatures ) ) ;

10 }

11

12 //compute all pairwise distances by feature maps

13 final int dim = featureMaps . size ( ) ; double [ ] [ ] matrix = **new **double [ dim ] [ dim ] ;

14 IDistanceMeasure similarity = **new **DistanceTanimoto ( ) ;

15    **for **( int i = 0; i < dim; i++) {

16       **for **( int j = i ; j < dim ; j++) {

17       matrix [ i ] [ j ] = similarity . getSimilarity (featureMaps . get ( i ), featureMaps . get ( j ));

18    }

19 }
